# «The Application of the New Law of Adoption and the Psychological Preparation of Prospective Stepparents»

**DOI:** 10.1192/j.eurpsy.2022.2288

**Published:** 2022-09-01

**Authors:** I. Farmakopoulou, K. Stavropoulou, N. Bari, N. Theodoropoulou, M. Theodoratou

**Affiliations:** 1 University of Patras, Humanistic Sciences, Patras, Greece; 2 University of Patras, Social Work, Patras, Greece; 3 Hellenic Open University, Humanistic Sciences, Patras, Greece

**Keywords:** Adoption, Emotions, Legal Framework for Adoption, Social Worker

## Abstract

**Introduction:**

The new adoption law, which was introduced in Greece in 2018, brought radical changes in adoption.

**Objectives:**

This presentation aimed to investigate the emotions of the prospective stepparents, based on the changes which were effectuated by the new law of adoption.

**Methods:**

This survey was conducted through mixed methodology. Quantitative research was addressed to investigate the emotions of the to prospective stepparents. Qualitative research was addressed to social workers and its goal was to depict their opinion about the new law and the prospective stepparents’ expressed emotions.

**Results:**

The findings of the survey have demonstrated that the stepparents had a variety of emotions that changed throughout the adoption process. The dominant feelings of parents at the first visit with the social worker were stress and anxiety. However, at the end of the training process, these feelings were replaced by confidence and impatience. This emotional rotation of prospective stepparents was confirmed in the social workers’ interviews. In addition, social workers interviews highlighted the importance of the amendment of the old law of adoption’s process.

**Conclusions:**

To sum up, this research has shown the importance of the new law and therefore the significance of its right application from all children’s placements in Greece. Nevertheless, it seems that due to the recent application of the new law, many placements have not yet complied with it and therefore there is urgent need for reinforcement of its application, according to the guidelines of the European Union that demanded gradual deinstitutionalization using the alternative types of child care.

**Disclosure:**

No significant relationships.

